# Is MRCP necessary to diagnose pancreas divisum?

**DOI:** 10.1186/s12880-019-0329-1

**Published:** 2019-04-29

**Authors:** Nino Bogveradze, Felix Hasse, Philipp Mayer, Christian Rupp, Christin Tjaden, Miriam Klauss, Hans-Ulrich Kauczor, Tim Frederik Weber

**Affiliations:** 1 Department of MRI, Research Institute of Clinical Medicine (Todua Clinic), 13 Tevdore mgvdlis St., 0112 Tbilisi, Georgia; 2 Department of Diagnostic and Interventional Radiology, Heidelberg University Hospital, INF 110, 69120 Heidelberg, Germany; 3 Department of Gastroenterology, Infectious Diseases, Intoxication, Heidelberg University Hospital, INF 410, 69120 Heidelberg, Germany; 4 Department of General, Visceral and Transplantation Surgery, Heidelberg University Hospital, INF 110, 69120 Heidelberg, Germany

**Keywords:** MRI, MRCP, TIRM, HASTE, Pancreas divisum

## Abstract

**Background:**

The purpose of this study is to compare the performance of three-dimensional magnetic resonance cholangiopancreatography (3D-MRCP) with non-MRCP T2-weighted magnetic resonance imaging (MRI) sequences for diagnosis of pancreas divisum (PD).

**Methods:**

This is a retrospective study of 342 consecutive patients with abdominal MRI including 3D-MRCP. 3D-MRCP was a coronal respiration-navigated T2-weighted sequence with 1.5 mm slice thickness. Non-MRCP T2-weighted sequences were (1) a coronal inversion recovery sequence (TIRM) with 6 mm slice thickness and (2) a transverse single shot turbo spin echo sequence (HASTE) with 4 mm slice thickness. For 3D-MRCP, TIRM, and HASTE, presence of PD and assessment of evaluability were determined in a randomized manner. A consensus read by two radiologists using 3D-MRCP, non-MRCP T2-weighted sequences, and other available imaging sequences served as reference standard for diagnosis of PD. Statistical analysis included performance analysis of 3D-MRCP, TIRM, and HASTE and testing for noninferiority of non-MRCP T2-weighted sequences compared with 3D-MRCP.

**Results:**

Thirty-three of 342 patients (9.7%) were diagnosed with PD using the reference standard. Sensitivity/specificity of 3D-MRCP for detecting PD were 81.2%/69.7% (*p* < 0.001). Sensitivity/specificity of TIRM and HASTE were 92.5%/93.9 and 98.1%/97.0%, respectively (p < 0.001 each). Grouped sensitivity/specificity of non-MRCP T2-weighted sequences were 99.8%/91.0%. Non-MRCP T2-weighted sequences were non-inferior to 3D-MRCP alone for diagnosis of PD. 20.2, 7.3%, and 2.3% of 3D-MRCP, TIRM, and HASTE, respectively, were not evaluable due to motion artifacts or insufficient duct depiction.

**Conclusions:**

Non-MRCP T2-weighted MRI sequences offer high performance for diagnosis of PD and are noninferior to 3D-MRCP alone.

**Trial registration:**

Not applicable.

## Background

Anatomic anomalies of the pancreas can be classified as either fusion anomaly (pancreas divisum), migration anomaly (annular pancreas, ectopic pancreas), or duplication anomaly (number or form variation). Pancreatic fusion and migration anomalies may result in a predisposition to specific pancreatic or peripancreatic diseases [1]. Pancreas divisum (PD) is the most common congenital anomaly, occurring in 4 to 14% of the population according to autopsy studies [1]. It is the result of a failure of the ventral and dorsal pancreatic anlagen to fuse during the fifth week of embryologic development. Consequently, the dorsal duct drains most of the pancreatic glandular tissue via the minor papilla [1]. PD is associated with acute and recurrent pancreatitis. The frequency of PD in patients with idiopathic pancreatitis is being reported to reach up to 50% [2, 3].

Endoscopic retrograde cholangiopancreatography (ERCP) was the modality of choice for diagnosing pancreas divisum, but is an invasive technique that is associated with radiation exposure and procedure-related complications such as post-ERCP-pancreatitis [4]. To reduce the ERCP complication rate, cannulation of the pancreatic duct is avoided if the biliary duct system is the focus of the examination (ERC).

Nowadays, magnetic resonance cholangiopancreatography (MRCP) is a well-established noninvasive and per se complication-free examination for many pancreaticobiliary conditions including PD that makes use of heavily T2-weighted pulse sequences to depict the fluid-containing pancreaticobiliary duct system [5]. Three-dimensional MRCP (3D-MRCP) using fast spin echo pulse sequences with (near) isotropic spatial resolution and synchronization to respiration represents the most elaborate MRCP technique currently used in clinical routine [5]. Despite synchronization to respiration, however, prolonged acquisition times of 3D-MRCP may lead to suboptimal image quality due to motion artifacts especially in non-cooperative patients, and limited anatomical coverage may hamper the diagnostic capability of the sequence [6–8].

The so-called one-stop-shop MRI combines dedicated MRCP sequence protocols with other routine T2-weighted imaging sequences such as half Fourier acquisition single shot turbo spin echo sequences (HASTE) and inversion recovery turbo spin echo sequences (TIRM) for complete abdominal imaging work-up. These T2-weighted imaging sequences provide high signal intensity of structures containing water-bound protons and allow for assessment of focal lesions and fluid collections but also of the pancreaticobiliary system. Each slice is being obtained in approximately one second so that these techniques are known to be relatively insensitive to motion artifacts [9]. [10].

The primary objective of this study was to test whether non-MRCP T2-weighted MRI sequences alone or in combination are noninferior to 3D-MRCP in the diagnosis of PD. Secondary objectives were to analyze sensitivity and specificity of non-MRCP T2-weighted MRI sequences and 3D-MRCP for the diagnosis of PD.

## Methods

### Study design

This is a retrospective single center exploratory study on patients referred to standard 3D-MRCP without secretin enhancement at our institution between October 2016 and August 2017. The study was approved by the institutional review board, and informed consent was waived. Inclusion criteria were age of at least 18 years and availability of MRCP as a part of the individuals’ standard abdominal MRI work-up. Exclusion criteria were history of pancreaticobiliary surgery (excluding cholecystectomy) and incomplete MRI protocol lacking one or more of the sequences regularly included in the exam (see below). For patients who received more than one MRI with 3D-MRCP during the study period, only the first examination was considered.

### Imaging

All MRI examinations were performed using a 1.5 T scanner (Magnetom AvantoFit, Siemens Healthcare, Erlangen, Germany). Patients fasted at least 6 h before the examination. Oral contrast and spasmolytic medication were not administered*.*

Technical parameters of 3D-MRCP and non-MRCP T2-weighted sequences are summarized in Table 1. 3D-MRCP was a three-dimensional respiration-navigated T2-weighted turbo spin echo sequence in coronal orientation with a slice thickness of 1.5 mm. Non-MRCP T2-weighted sequences were: First, a multi-breathhold TIRM in coronal orientation with a slice thickness of 6 mm. Second, a multi-breathhold HASTE in transverse orientation with a slice thickness of 4 mm.

**Table 1:** Technical parameters of 3D-MRCP and non-MRCP T2-weighted sequences

	3D-MRCP	TIRM	HASTE
Orientation	Coronal	Coronal	Transverse
Voxel size [mm]	0.9 × 0.9 × 1.5	1.0 × 1.0 × 6.0	1.2 × 1.2 × 4.0
Distance factor	50%	10%	10%
TR [ms]	2000	1000	1400
TE [ms]	686	79	93
Parallel imaging (acceleration factor)	GRAPPA (3)	GRAPPA (2)	none

Other sequences in the MRI protocol were: First, a pre-contrast T1-weighted three-dimensional fast low angle shot volumetric interpolated breath-hold examination with controlled aliasing in parallel imaging (VIBE-CAIPIRINHA) and Dixon technique for fat suppression in transverse orientation (slice thickness, 3 mm). Second, pre- and post-contrast (arterial phase, portal-venous phase, delayed phase) VIBE-CAIPIRINHA with spectral fat suppression in transverse orientation (slice thickness, 3 mm). Gadopentetate-Dimeglumine (Gadovist, Bayer Healthcare, Berlin, Germany) was administered at a dose of 0.1 mmol/kg body weight and a flow rate of 2 ml/s, followed by a 30 ml saline flush using a power injector.

### Image analysis

Images were analyzed using a picture archiving and communication system. 3D-MRCP, TIRM, and HASTE were assessed for presence of PD independently in a randomized manner by a single radiologist (reader 1). A panel consensus on presence of PD reached by two radiologists (reader 1 and reader 2) using the combination of 3D-MRCP, TIRM, HASTE, and the above mentioned other imaging sequences together was defined as the standard of reference. Reader 1 and reader 2 had 4 years and 12 years of experience in abdominal imaging, respectively, and were blinded to clinical information. Disagreement between two radiologists was managed by discussion.

PD was deemed to be present when the dorsal pancreatic duct crossed the common bile duct, opened into the duodenum via the minor papilla, and was separated from a smaller ventral duct.

If assessment of the pancreatic duct anatomy was not possible, it was assessed whether this was attributable either to motion artifacts or to insufficient duct visibility on images not compromised by motion artifacts. Image quality was determined by assessing the general magnitude of motion artifacts present on each image series and was subjectively scored using a Likert scale ranging from 0 (no artifacts) to 3 (severe artifacts).

### Statistical analysis

All statistics were calculated using IBM SPSS Statistics release 23 (Statistical Package for the Social Sciences, IBM Corporation, Armonk, USA). The diagnostic performance of 3D-MRCP, TIRM, and HASTE for diagnosis of PD was determined in comparison with the standard of reference including sensitivity, specificity, and negative predictive value. A noninferiority test was performed to analyze similarity between 3D-MRCP on the one hand and HASTE and TIRM on the other hand for diagnosis of PD. Noninferiority was assumed if the sensitivity of the test method (non-MRCP T2-weighted sequences, *P*_T_) was not worse than the sensitivity of the comparator method (3D-MRCP, *P*_C_) by using a 1-sided test with a noninferiority margin of Δ. The primary hypothesis can be stated as *H*_0_: *P*_T_ − *P*_C_ ≤ −Δ. Noninferiority of *P*_T_ to *P*_C_ is inferred when the lower bound of either confidence interval (CI) is above the noninferiority margin (−Δ) [11]. The margin Δ was defined to be 0.5 on the basis of the population and was confirmed by clinical estimates from prior research and institutional pilot data. Image quality assessments were compared between sequence protocols using paired t-tests.

## Results

### Study population

During the study period, in 436 consecutive patients abdominal MRI including 3D-MRCP was performed. Eighty-eight patients had to be excluded due to history of pancreatobiliary surgery. Six patients had to be excluded due to incomplete MRI protocol. Thus, in total 342 patients were available for analysis (sex, 170 females; mean age, 51 ± 16 years). MRI with 3D-MRCP was performed for a variety of clinical indications, which are summarized in Table 2. Pancreatic findings observed in the study patients are summarized in Table 3.

**Table 2:** Clinical indications for MRI with MRCP

	Extrapancreatic tumor	Extrapancreatic non-oncological disease	Intrapancreatic tumor	Intrapancreatic inflammatory disease	Other
n	20	69	112	137	4

**Table 3:** Pancreatic findings

	Unremarkable pancreas	Cystic lesions ≥ 10 mm	Cystic lesions < 10 mm	Signs of pancreatitis	Other
n	169	59	74	63	10

### Acquisition times

Mean acquisition time of 3D-MRCP was 176 ± 66 s. Acquisition times per sequence protocol design were 55 s and 74 s for TIRM and HASTE, respectively.

### Assessment of PD

Thirty-three of 342 patients (9.7%) were diagnosed with PD using the standard of reference.

Sensitivity and specificity of 3D-MRCP for diagnosing PD was 81.2 and 69.7% (*p* < 0.001). Sensitivity and specificity of TIRM for diagnosing PD was 92.5 and 93.9% (p < 0.001). Sensitivity and specificity of HASTE for diagnosing PD was 98.1 and 97.0% (*p* < 0.001). Grouped sensitivity and specificity of the combination of non-MRCP T2-weighted sequences was 99.8 and 91.0%. The negative predictive value of the combination of non-MRCP T2-weighted sequences was 99.9%.

**Fig. 1:**
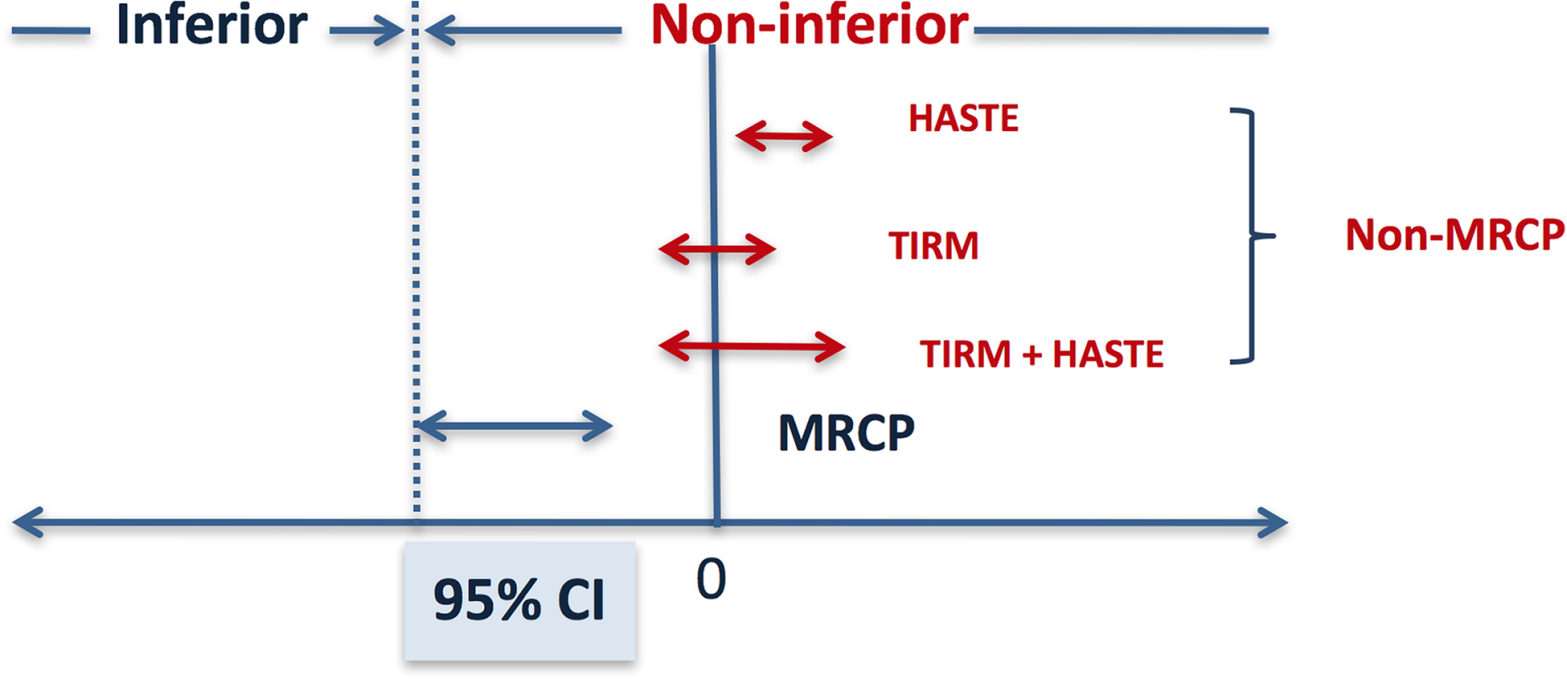
Non-inferiority test comparing 3D-MRCP with TIRM and HASTE

Both TIRM alone (lower bound of 95% CI -0.03) and HASTE alone (lower bound of 95% CI 0.3) as well as the combination of TIRM and HASTE (lower bound of 95% CI 0.06) were noninferior to 3D-MRCP (lower bound of 95% CI -0.3) for diagnosis of PD (Fig. 1).

### Image quality

The mean general magnitude of motion artifacts was scored with 1.07 ± 0.86, 0.03 ± 0.19, and 0.10 ± 0.33 for 3D-MRCP, TIRM, and HASTE. The artifact scoring was better for both TIRM and HASTE than for 3D-MRCP (p < 0.001, each).

Assessment of pancreatic duct orifice anatomy was not possible in 20.2% (69/342), 7.3% (25/342), and 2.3% (8/342) of 3D-MRCP, TIRM, and HASTE, respectively. This was due to motion artifacts in 9.4% (32/342), 0.3% (1/342), and 0.0% (0/342) and due to insufficient duct depiction in 10.8% (37/342), 7.0% (24/342), and 2.3% (8/342) of 3D-MRCP, TIRM, and HASTE, respectively.

**Fig. 2:**
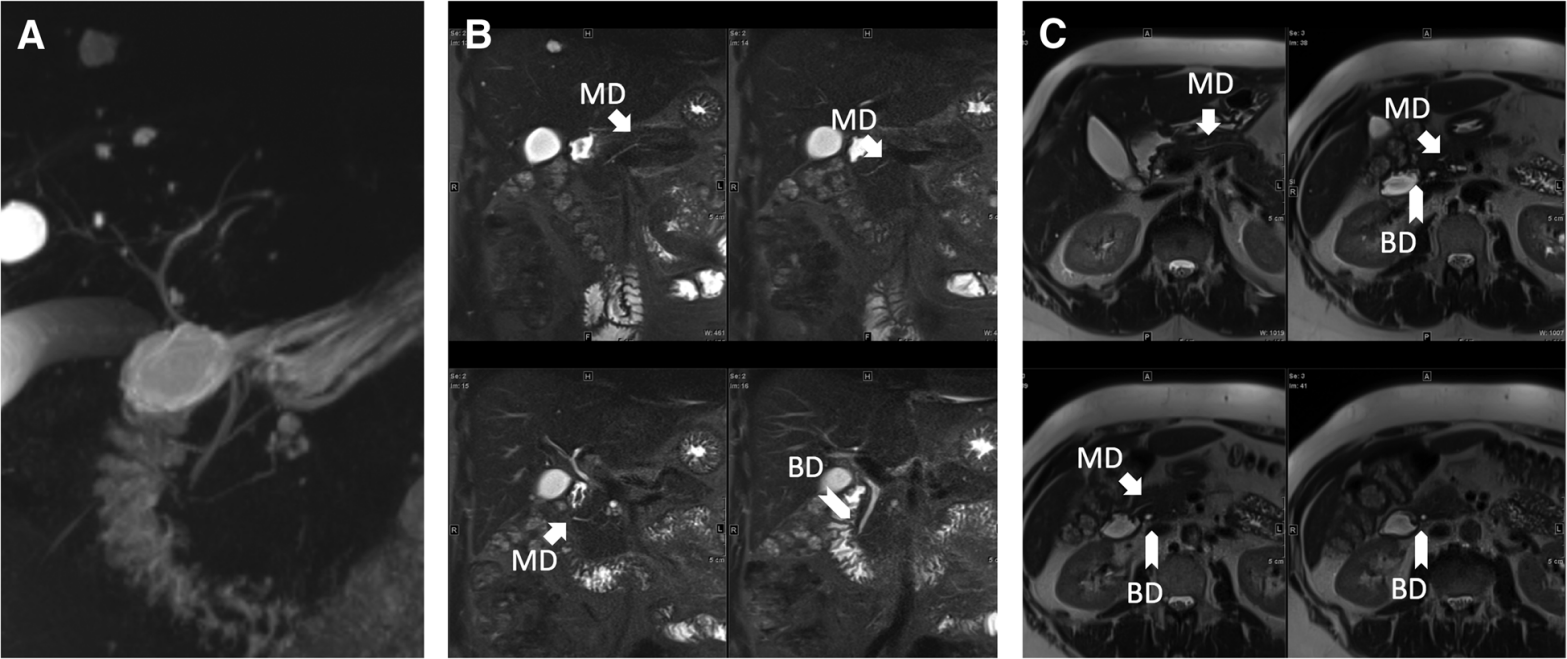
Pancreas divisum in 3D-MRCP. (**a**), TIRM (**b**), and HASTE (**c**). 3D-MRCP with good overall image quality and TIRM and HASTE show the main pancreatic duct (MD) crossing the bile duct (BD) cranially and opening via the minor papilla

**Fig. 3:**
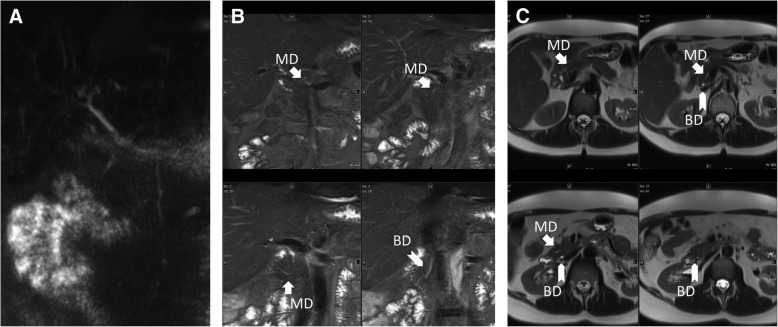
Pancreas divisum in 3D-MRCP. (**a**), TIRM (**b**), and HASTE (**c**). 3D-MRCP has poor overall image quality and does not show pancreas divisum. TIRM and HASTE clearly indicate presence of PD

Figures 2 and 3 show representative examples of PD diagnosis in a patient with 3D-MRCP without artifacts (Fig. 2) and in a patient with 3D-MRCP with severe artifacts (Fig. 3).

## Discussion

The results of our study indicate that non-MRCP T2-weighted sequences frequently included in routine abdominal MRI protocols not primarily focusing on pancreatic duct visualization have high diagnostic performance for diagnosis of PD, are noninferior to dedicated 3D-MRCP concerning PD diagnosis, and are less prone to motion artifacts than 3D-MRCP.

PD is the most common anatomic variant of the pancreas. The frequency of PD observed in our study is comparable to previous reports [12]. PD can be diagnosed on imaging studies when the dorsal pancreatic duct crosses the common bile duct anteriorly with a constant caliber, opens into the duodenum via the minor papilla, and is separated from a smaller ventral duct [13].

Aside from PD there are other anatomic variants in which pancreatic duct drainage via the minor papilla can be seen. These include a patent minor papilla and incomplete PD [13]. Opposed to the PD definition mentioned above and used in this study, a patent minor papilla is considered to be present if the dominant pancreatic duct drains via the major papilla and a smaller pancreatic duct drains via the minor papilla. In incomplete PD, there is a visible connection between the dominant duct that drains via the minor papilla and the smaller ventral duct that drains via the major papilla. Data indicate that clinical presentation and symptom occurrence rate of incomplete PD and complete PD are similar, so that we did not aim to differentiate between these two closely related variants [14, 15].

The non-MRCP sequences used in our study to assess presence of PD are standard T2-weighted sequences generally recommended to be part of abdominal MRI [9]. T2-weighted imaging sequences are of special use for characterization of fluid containing structures. Some studies have shown their value especially for pancreatic cystic lesions. Pozzi-Mucelli et al. have demonstrated that an abbreviated pancreas MRI only including a transverse and coronal HASTE with a slice thickness of 4 mm is similar to a comprehensive protocol including 3D-MRCP for diameter assessment of cystic pancreatic masses [16]. Macari et al. reported that using a MRI protocol based on HASTE and TIRM sequences one could abstain from contrast administration for follow-up of pancreatic cystic lesions [17]. Chalazonitis et al. describe one case of incomplete pancreas divisum, in that findings were more evident on HASTE images than on dedicated MRCP images [18].

The diagnostic performance of MRCP for detection of PD reported in the literature varies and is in part depending on the MRCP technique used. In an older study by Bret et al., MRCP was able to detect PD in all patients that were diagnosed with PD in ERCP [19]. In another older study by Ueno et al., sensitivity of MRCP for PD diagnosis was below 50% [20]. Newer studies report on sensitivities of MRCP for PD diagnosis that lie in between of these values, e.g. Carnes et al. [21], Mosler et al. [6] or Kushnir et al. [22]. Carnes et al. discussed that suboptimal MRI techniques and reader inexperience are relevant contributing factors to miss PD diagnosis [21]. Although synchronized to respiration, our data show that 3D-MRCP is still prone to motion artifacts in clinical routine in patients with limited capability for shallow constant breathing.

Other frequently used MRCP techniques are two-dimensional sequence protocols that can be performed as a thick slab acquisition with slice thicknesses from 40 to 60 mm or as multiple thinner slices (less than 5 mm) during breath-holding. Thick slab MRCP represents a summation image of the scanned volume that hampers spatial mapping of findings and, thus, may suggest anatomical relationships that may not be present. Although two-dimensional MRCP sequences are faster than 3D-MRCP and have a role in assessing pancreatic duct anatomy, thick slab MRCP is not part of the MRI protocol used in this study, because its diagnostic value in evaluating pancreaticobiliary disease can be discussed controversially and was frequently shown to be inferior to 3D-MRCP [23–27].

Negative oral contrast agents are suggested to be used especially in thick slab MRCP to eliminate signal from overlapping fluid-containing bowel [10] or may improve the informative value of maximum intensity projections (MIP) reconstructed from 3D-MRCP. Since in our study not MIPs but the original slices of 3D-MRCPs were analyzed, we believe that not using negative oral contrast does not impact negatively on duct assessment.

Publications have suggested that PD diagnosis by MRCP can be facilitated after intravenous administration of secretin to stimulate pancreatic excretion [28]. However, data on secretin-enhanced MRCP concerning PD diagnosis are ambiguous [21]. Thus, at our institution secretin is only used in special cases when functional information on pancreatic juice secretion is needed (pancreatic insufficiency, papilla stenosis).

### Limitations

There are limitations to our study. ERCP as the historical gold standard for assessment of pancreaticobiliary duct anatomy was not available in our patients, and we used a combination of all available image series to define the ground truth. Nowadays, the mainstay of endoscopic procedures is depicting the pancreatic and biliary system in patients with high pretest probability for necessity of a simultaneous endoscopic therapeutic procedure and are, thus, frequently limited to ERC to reduce complication rates. Taking this into account, MRCP techniques may be considered the de facto gold standard for assessing the pancreatic duct anatomy. Endosonography represents an endoscopic alternative to ERCP for PD assessment [29]. In the absence of a non-invasive reference standard for PD diagnosis different from MRI, all available imaging sequences including the investigational sequences were used to determine ground truth. This may lead to an incorporation bias resulting in potential overestimation of the diagnostic test’s accuracy. We only performed a single-reader analysis for assessment of PD and a two-person consensus panel defined the reference standard. Thus, information on reproducibility and inter-rater variability of our data cannot be inferred. As the number of cases positive for PD in our study is low, the results should be confirmed in a larger prospective trial.

## Conclusion

Non-MRCP T2-weighted MRI sequences usually included in standard abdominal MRI protocols are noninferior to 3D-MRCP alone in regards of correctly diagnosing PD. It may not be necessary to perform 3D-MRCP to assess pancreatic duct orifice anatomy.

## Abbreviations

CI: Confidence interval
CT: Computed tomography
ERCP: Endoscopic retrograde cholangiopancreatography
HASTE: Half-fourier single shot turbo spin echo
MIP: Maximum intensity projection
MRCP: Magnetic resonance cholangiopancreatography
MRI: Magnetic resonance imaging
PD: Pancreas divisum
TIRM: Turbo inversion recovery with magnitude display
VIBE-CAIPIRINHA: Volumetric interpolated breath-hold examination with controlled aliasing in parallel imaging

## References

[1] Borghei P, Sokhandon F, Shirkhoda A, Morgan DE (2013). Anomalies, anatomic variants, and sources of diagnostic pitfalls in pancreatic imaging. Radiology..

[2] Fogel EL, Toth TG, Lehman GA, DiMagno MJ, DiMagno EP (2007). Does endoscopic therapy favorably affect the outcome of patients who have recurrent acute pancreatitis and pancreas divisum?. Pancreas.

[3] Bernard JP, Sahel J, Giovannini M, Sarles H (1990). Pancreas divisum is a probable cause of acute pancreatitis: a report of 137 cases. Pancreas.

[4] Morales SJ, Sampath K, Gardner TB (2018). A review of prevention of post-ERCP pancreatitis. Gastroenterol Hepatol (N Y).

[5] Griffin N, Charles-Edwards G, Grant LA (2012). Magnetic resonance cholangiopancreatography: the ABC of MRCP. Insights Imaging.

[6] Mosler P, Akisik F, Sandrasegaran K, Fogel E, Watkins J, Alazmi W (2012). Accuracy of magnetic resonance cholangiopancreatography in the diagnosis of pancreas divisum. Dig Dis Sci.

[7] Patel KR, Parikh J, Hussain Z, Gourtsoyianni S, Griffin N (2015). The diagnostic and technical quality of magnetic resonance cholangiopancreatography (MRCP). Clin Radiol.

[8] Kim JH, Hong SS, Eun HW, Han JK, Choi B-I (2012). Clinical usefulness of free-breathing navigator-triggered 3D MRCP in non-cooperative patients: comparison with conventional breath-hold 2D MRCP. Eur J Radiol.

[9] Guglielmo FF, Kania LM, Ahmad HM, Roth CG, Mitchell DG (2016). Interpreting body MRI cases: what you need to know to get started. Abdominal Radiol.

[10] Matos C, Cappeliez O, Winant C, Coppens E, Devière J, Metens T (2002). MR imaging of the pancreas: a pictorial tour. RadioGraphics.

[11] Ahn S, Park SH, Lee KH (2013). How to demonstrate similarity by using noninferiority and equivalence statistical testing in radiology research. Radiology.

[12] Bülow R, Simon P, Thiel R, Thamm P, Messner P, Lerch MM (2014). Anatomic variants of the pancreatic duct and their clinical relevance: an MR-guided study in the general population. Eur Radiol.

[13] Delhay M, Matos C, Deviere J (2001). Acute relapsing pancreatitis. Congenital variants: diagnosis, treatment, outcome. JOP.

[14] Kim MH, Lee SS, Kim CD, Lee SK, Kim HJ, Park HJ (2001). Incomplete pancreas divisum: is it merely a normal anatomic variant without clinical implications?. Endoscopy.

[15] Kamisawa T, Tu Y, Egawa N, Tsuruta K, Okamoto A (2006). Clinical implications of incomplete pancreas divisum. JOP.

[16] Pozzi-Mucelli RM, Rinta-Kiikka I, Wünsche K, Laukkarinen J, Labori KJ, Ånonsen K (2017). Pancreatic MRI for the surveillance of cystic neoplasms: comparison of a short with a comprehensive imaging protocol. Eur Radiol.

[17] Macari M, Lee T, Kim S, Jacobs S, Megibow AJ, Hajdu C (2009). Is gadolinium necessary for MRI follow-up evaluation of cystic lesions in the pancreas? Preliminary results. Am J Roentgenol.

[18] Chalazonitis NA, Lachanis BS, Laspas F, Ptohis N, Tsimitselis G, Tzovara J (2008). Pancreas divisum: magnetic resonance cholangiopancreatography findings. Singap Med J.

[19] Bret PM, Reinhold C, Taourel P, Guibaud L, Atri M, Barkun AN (1996). Pancreas divisum: evaluation with MR cholangiopancreatography. Radiology..

[20] Ueno E, Takada Y, Yoshida I, Toda J, Sugiura T, Toki F (1998). Pancreatic diseases: evaluation with MR cholangiopancreatography. Pancreas.

[21] Carnes ML, Romagnuolo J, Cotton PB (2008). Miss rate of pancreas divisum by magnetic resonance cholangiopancreatography in clinical practice. Pancreas.

[22] Kushnir VM, Wani SB, Fowler K, Menias C, Varma R, Narra V (2013). Sensitivity of endoscopic ultrasound, multidetector computed tomography, and magnetic resonance cholangiopancreatography in the diagnosis of pancreas divisum: a tertiary center experience. Pancreas.

[23] Lavdas E, Vlychou M, Arikidis N, Kapsalaki E, Roka V, Arvanitis DL (2013). How reliable is MRCP with an SS-FSE sequence at 3.0 T: comparison between SS-FSE BH and 3D-FSE BH ASSET sequences. Clin Imaging.

[24] Palmucci S, Mauro LA, Coppolino M, Musumeci AG, Foti PV, Milone P (2010). Evaluation of the biliary and pancreatic system with 2D SSFSE, breath- hold 3D FRFSE and respiratory-triggered 3D FRFSE sequences. Radiol Med.

[25] Kinner S, Dechêne A, Ladd SC, Zöpf T, Maldonado de Dechêne E, Gerken G (2010). Comparison of different MRCP techniques for the depiction of biliary complications after liver transplantation. Eur Radiol.

[26] Kinner S, Steinweg V, Maderwald S, Radtke A, Sotiropoulos G, Forsting M (2014). Comparison of different magnetic resonance cholangiography techniques in living liver donors including Gd-EOB-DTPA enhanced T1-weighted sequences. PLoS One.

[27] Kang SK, Heacock L, Doshi AM, Ream JR, Sun J, Babb JS (2017). Comparative performance of non-contrast MRI with HASTE vs. contrast-enhanced MRI/3D-MRCP for possible choledocholithiasis in hospitalized patients. Abdominal Radiol.

[28] Manfredi R, Costamagna G, Brizi MG, Spina S, Maresca G, Vecchioli A (2000). Pancreas divisum and “santorinicele”: diagnosis with dynamic MR cholangiopancreatography with secretin stimulation. Radiology..

[29] Sahakian AB, Aslanian HR (2014). Diagnosis of pancreas Divisum using linear-Array Endosonography. Video J Enc GI Endoscopy.

